# Influence of Pre-Fermentation Treatments on Wine Volatile and Sensory Profile of the New Disease Tolerant Cultivar Solaris

**DOI:** 10.3390/molecules201219791

**Published:** 2015-12-03

**Authors:** Shujuan Zhang, Mikael Agerlin Petersen, Jing Liu, Torben Bo Toldam-Andersen

**Affiliations:** 1 Department of Food Science, Faculty of Science, University of Copenhagen, Rolighedsvej 30, Frederiksberg C DK-1958, Denmark; map@food.ku.dk (M.A.P.); ljing@food.ku.dk (J.L.); 2 Department of Plant and Environmental Science, Faculty of Science, University of Copenhagen, Højbakkegård Alle 13, Tåstrup DK-2630, Denmark; tbta@plen.ku.dk

**Keywords:** maceration, skin fermentation, whole cluster press, volatile compounds, sensory analysis, PLS

## Abstract

Solaris is a new disease tolerant cultivar increasingly cultivated in cool climate regions. In order to explore the winemaking processes’ potential to make different styles of Solaris wines, the effects of different pre-fermentation treatments (direct press after crushing, whole cluster press, cold maceration, and skin fermentation) on the volatile profile, chemical, and sensory properties of Solaris wines were investigated. Cold maceration treatment for 24 h and fermentation on skin led to wines with lower acidity and higher glycerol and total polyphenol indexes. Sensory analysis showed that cold maceration enhanced “apricot” and “apple” flavor while skin fermentation gave rise to increased “rose” and “elderflower” flavor. The PLS regression model revealed that fruity flavor of cold macerated wines was related to a combination of esters while β-damascenone and linalool were correlated to the “rose” and “elderflower” flavor. This study provides information about pre-fermentation techniques that allowed the possibility of obtaining wines with different styles.

## 1. Introduction

The style of a typical wine strongly depends on various factors such as cultivar, year of harvest, winemaking practices, and climate conditions, of which the winemaking process is one of the most important factors. Pre-fermentation treatments (e.g., acid and sugar adjustment) are critical in cool and cold climate regions due to the fact that the grapes may not have ripened optimally. In addition, the presence and concentration of wine aroma components are significantly influenced by the applied pre-treatment techniques, as a high amount of aroma precursors are located in the grape skin and pulp. To enhance and optimize extraction of flavor components and precursors, cold maceration (CM) which refers to the release of components from the pomace (seeds, skins, and pulp) after crushing is often applied in white wine making. This process strongly determines the final styles of wine produced [1,2,3,4,5]. Extended extraction can also be achieved by skin fermentation (SF) in order to make fully-flavored and complex styles of white wine [6]. Some important volatiles, such as C-6 alcohols and aldehydes are derived originally from the solid parts of grape berry and, therefore, promoted by increased extraction through maceration [7,8,9]. Additionally, maceration enhances the concentration of many non-volatiles such as polyphenols which in turn result in more mouthfeel and in higher levels of antioxidants in the final wine [10,11,12]. Sensory assessments have demonstrated that these pre-treatments confer fresh properties to white wines [5], enhance flavor intensity [9,13], and improve fruity and floral flavor [14,15] in the wines, while may also add bitterness to white wines.

However, maceration practices are largely cultivar dependent, and vary with vineyard and vintage conditions [16]. The influence of maceration on the amount of most volatiles depends on temperature and duration [17,18]. Low maceration temperature is normally employed in white wine making, while duration may vary from wine to wine. For instance, Selli *et al.* [2] investigated “Muscat” wines produced with 6 h of maceration at 15 °C and found higher quality than after 12 h. In another study, the concentration of terpenes, norisoprenoids, and benzene compounds in “Albillo” wines was considerably enhanced by CM for 15 and 23 h [5]. Furthermore, the total aroma concentration increased with the extension of CM time in “Chardonnay” wines [19]. However, no differences were detected for most free volatiles between macerated and non-macerated “Listán blanco” wines [20]. In this sense, the development of suitable winemaking techniques for specific cultivars is crucial in a certain location/region.

“Solaris” is a new disease tolerant cultivar grown in northern Europe with advantages such as stable yields and reliable berry ripening despite the cool climate [21]. An average of 97 days is required to fully ripen the berries and the typical yield is 0.5 kg/m^2^ (from year 2007 to 2014). “Solaris” has an average must of 20.9 °Brix and about 9.4 g/L titratable acidity and is considered to be ripe every year over the last 10 years in Denmark. It is largely predominant in Denmark, England, Southern Sweden and other regions in Northern Europe (The Netherlands, Belgium, North Germany, and Poland), producing a good single varietal wine. Selecting the best cultivars is crucial, especially in marginal and cool climate regions, to explore the potential of making different styles of wine, specific winemaking techniques for the cultivar need to be developed. However, to date, little is known about the potential of different wine style production from this newly-released cultivar, especially on the impact of different pre-fermentation processes on Solaris wine quality. Therefore, the overall aim of this study was to investigate the influence of different pre-fermentation treatments on volatile profile, chemical parameters, and sensory features of “Solaris” white wines.

## 2. Results and Discussion

In order to reduce the effect of factors other than the maceration treatments, grapes from the same vineyard were used. The overall ripeness of “Solaris” grapes in 2011 were at a good level with 21.1 °Brix in the direct pressed must. This density means that the wine has the potential to obtain an alcohol level considered optimal for a white wine style from a cool region as Denmark, being not too alcohol dominant while grapes were still ripe enough to have a good flavor potential. Details of pre-fermentation treatments are shown in Table 1.

**Table 1:** Pre-fermentation treatments applied in the study in “Solaris” white wines.

Wine Code	Pre-Fermentation Treatments
DP	Direct press after crushing
WC	Whole cluster press
6H_CM	6 h cold maceration
24H_CM	24 h cold maceration
6H_CM + SF	6 h cold maceration + 30 h skin fermentation
24H_CM + SF	24 h cold maceration + 30 h skin fermentation

### 2.1. Impact of Pre-fermentation Treatments on Wine Chemical Parameters

The pre-fermentative treatments WC and DP resulted in low levels of potassium, yeast assimilable nitrogen (YAN) in the juice and low pH in the wine (Table 2), in accordance with general oenology literature [16]. CM treatment increased YAN from moderate in DP sample to high level in 6H_CM and 24H_CM samples. Additionally, CM increased pH but addition of SF during early fermentation did not result in any further pH increase (Table 2). However, when measured in the young wine titratable acidity and especially tartaric acid levels were significantly influenced by the time of skin contact. The lower tartaric acid levels and higher pH observed after 24H_CM + SF could be due to the higher potassium (Table 2) or other cations extracted from the grape skins during skin fermentation [22], which made further deacidification unnecessary in this wine. As deacidification has been shown to have no or very limited effect on the volatile profile of wine (unpublished results from our lab), the acidity effects of the treatments were balanced by deacidification up to 2.50 g/L on the rest of experimental wines.

Table 3 shows that the later cold stabilization further reduced and stabilized acidity in the final wines. The levels of ethanol values ranged from 12.2% to 12.9% with a tendency of increasing levels after CM and SF. Additionally, the total polyphenol index was higher in the final wines with longer skin contact (24H_CM + SF wine). The present result was in line with previous studies [10,11,23]. Volatile acidity was generally low indicating a reductive and successful winemaking process. In addition, the concentration of glycerol was around 6 g/L in all wines matching the level of alcohol produced by the yeast. The concentration is above the taste threshold level for glycerol (5.2 g/L in wine) and may, thus, positively contribute to the smoothness of the final wines [24].

**Table 2:** Chemical parameters of “Solaris” juice and young wines after completing of fermentation and levels of deacidification with calcium carbonate.

Parameters	Pre-fermentation Treatments					
	DP	WC	6H_CM	24H_CM	6H_CM + SF	24H_CM + SF
Juice						
°Brix	21.1	21.2	21.3	21.8	-	-
Potassium (in juice, g/L)	0.71 ^a,b^	0.52 ^b^	0.87 ^a,b^	0.94 ^a^	-	-
Ammonia (mg/L)	107	109	108	113	-	-
α-Amino nitrogen (mg/L)	188 ^b^	184 ^b^	238 ^a^	233 ^a^	-	-
YAN	276 ^b^	274 ^b^	326 ^a^	327 ^a^	-	-
Wine						
pH	3.18 ^c^	3.09 ^d^	3.31 ^b^	3.40 ^a^	3.36 ^a^	3.43 ^a^
Tartaric acid (g/L)	5.33 ^b^	5.52 ^a^	4.69 ^c^	4.14 ^d^	3.69 ^e^	2.97 ^f^
Titratable acidity (g/L)	9.57 ^b^	10.2 ^a^	8.91 ^c^	8.36 ^d^	8.40 ^d^	7.93 ^e^
Deacidification (g/L)	2.50	2.00	1.00	1.00	1.00	-

Different letters in the same rows indicates significant differences according to Tukey’s HSD test (*p* < 0.05); YAN was estimated by using formular [YAN] = 0.8225 × [ammonia] + [α-amino nitrogen].

**Table 3:** Chemical parameters of the final “Solaris” white wines.

Parameters	Wines					
	DP	WC	6H_CM	24H_CM	6H_CM + SF	24H_CM + SF
Ethanol (% *v*/*v*)	12.2 ^c^	12.5 ^b^	12.4 ^b^	12.7 ^a^	12.8 ^a^	12.9 ^a^
pH	3.43 ^a,b^	3.23 ^b^	3.41 ^b^	3.48 ^a^	3.51 ^a^	3.37 ^b^
Titratable acidity (g/L)	6.40 ^c^	7.58 ^a^	6.80 ^b^	6.44 ^c^	6.20 ^c^	6.77 ^b^
Volatile acid (g/L)	0.180 ^a,b^	0.190 ^a,b^	0.270 ^a^	0.270 ^a^	0170 ^a,b^	0.150 ^b^
Tartaric acid (g/L)	1.81 ^a,b^	2.10 ^a^	2.31 ^a^	1.34 ^a,b^	1.00 ^b^	1.00 ^b^
Malic acid (g/L)	3.21 ^d^	3.35 ^c,d^	3.56 ^b,c^	3.60 ^b,c^	3.70 ^a,b^	3.95 ^a^
Fructose (g/L)	0.640 ^b^	0.600 ^b^	0.630 ^b^	1.33 ^a^	0.600 ^b^	0.540 ^b^
Total polyphenol index	12.6 ^c^	12.2 ^b,c^	18.5 ^b^	16.3 ^b,c^	18.5 ^a,b^	21.9 ^a^
Glycerol (g/L)	5.95 ^b^	6.23 ^b^	6.09 ^a,b^	6.11 ^a,b,c^	5.74 ^a,b,c^	6.60 ^a^
Reducing sugar (g/L)	0.690 ^d^	0.960 ^c^	0.840 ^c^	1.47 ^a^	0.92 ^c,d^	1.19 ^b^

Different letters in the same rows indicates significant differences according to Tukey’s HSD test (*p* < 0.05).

### 2.2. Impact of Pre-Fermentation Treatments on Volatile Profile in Wine

In total, 71 volatile compounds, comprising 35 esters, 16 alcohols, seven aldehydes, eight terpenes, two ketones, two sulfur compounds, and one C_13_-norisoprenoid, were quantified in the wines. Table 4 shows concentrations of volatile compounds and their sensory descriptions. Thresholds and odor activity values (OAV) are also listed in the table. OAV allows estimation of the contribution of each volatile compound to wine aroma. It was determined by dividing the concentration by the odor threshold value of the compound reported in wine or matrix similar to wine, otherwise in water if not available in wine. To take the uncertainty of the calculated OAVs and the synergetic or additive impact of several volatile compounds into account, it was assumed that aroma compounds having an OAV of 0.1 or higher might be important in wine aroma.

Esters are both synthesized by the grapes and arise from the alcoholic fermentation. Esters are important in wine flavor due to their pleasant fruity odor. In this work, esters represent the most abundant group of volatiles detected and quantified in the wines, and nine might contribute to wine aroma directly due to their relatively high OAVs (Table 4, compounds in bold). CM and SF treatments had various impacts on the individual esters. Among nineteen identified ethyl esters, eight had OAV > 0.1 in all samples and among these, four were significantly influenced by the applied treatments. For example, the concentration of ethyl decanoate was significantly higher in 24H_CM wine than the rest of wines. Ethyl decanoate has been previously reported to provide fruity aroma to white wines [13]. CM and SF pre-treatments did not enhance the formation of ethyl esters of branched acids, for instance, ethyl 2-methylpropanoate, ethyl 2-methylbutanoate, and ethyl 3-methylbutanoate. This could be due to the high concentrations of amino nitrogen in CM wines. Ethyl 3-methylbutanoate exhibited the highest OAV (2.6) in WC wine. Ethyl 3-methylbutanoate has also been reported as an important odorant in Croatian “Rhine Riesling” wine [25].

The acetate esters, generated by the reaction of acetyl-CoA with higher alcohols from degradation of amino acids or fatty acids, were generally not significantly affected by CM and SF treatments. Only three acetates had OAV > 0.1 and all of them were not affected by the treatments. However, a slightly higher concentration of acetates was observed in 24H_CM wine. Dennis *et al.* [26] found that the content of an acetate ester increased with the pre-fermentation concentration of the corresponding precursor in a model must. Our results suggest a similar relationship between benzyl acetate and its precursor benzyl alcohol, as both increased in parallel in CM and SF wines (Table 4).

With respect to other esters (fatty acid esters of higher alcohols), the amount of methyl hexanoate, methyl octanoate, methyl decanoate, and methyl salicylate increased by CM and SF treatments. However, these esters are most probably not important to the wine aroma as all of them were present at levels far below their individual odor thresholds.

The influence of the maceration process on the concentration of esters depends on many factors, for instance, temperature [27], and grape cultivar [28] and it is difficult to draw any general conclusions. The amount of methyl octanoate was increased by CM treatment in this study, whereas the opposite effect was revealed in “Cabernet Sauvignon” wines [15].

Alcohols are the second major group of volatile components in “Solaris” wines. Higher alcohols generally have characteristic pungent odor and give complexity to wine flavor, which in turn influences the character of wines. They are the main fermentation derived products by the yeast via sugar catabolism or decarboxylation and deamination of amino acids [29]. As can be seen in Table 4, 3-methyl-1-butanol and 2-phenylethanol (OAV 2.4) were by far the most predominant by amount among the 11 identified higher alcohols. Despite their very high concentrations, the sensory impact was minor due to high sensory thresholds. It should be noted that the quantification of 3-methyl-1-butanol is imprecise due to overloaded peaks. A high initial nitrogen concentration generally results in decreased production of higher alcohols related to amino acid production [30]. In the present work, the amount of 2-methylpropanol, 1-propanol, and 2-phenylethanol decreased with increasing skin contact could be correlated the increased amount of YAN in the must with longer maceration (Table 2). However, a significant increase of the amount of 2-phenylethanol was observed in 24H_CM + SF wines. One possible reason could be that extensive skin contact increased the availability of some amino acids contributing to the biosynthesis of 2-phenylethanol which occurs preferentially in berry skin as suggested by Slegers *et al.* [31]. Nevertheless, its concentration was above threshold in all wines which might not relate to the sensory variations among studies wines.

The concentration of benzyl alcohol increased significantly with the CM and SF treatments, this is likely to be due to an increased extraction of glycosylated precursors [32,33].

C-6 compounds are originally present in crushed grape must, resulting from the enzymatic oxidation of grape polyunsaturated fatty acids through the lipoxygenase pathway [29]. In this sense, unsurprisingly, WC wine contained the lowest content of C-6 alcohols (Table 4). The 24 h CM generally favored the yield of C-6 alcohols in our study and this was in agreement with studies in “Albillo” and “Narince” wines [2,5], as well as “Chardonnay”, “Muscat” and “Cabernet Sauvignon” wines [8,18,28]. In this study, however, these C-6 alcohols exhibited concentrations lower than their odor threshold, and therefore may not be sensed in the aroma of the macerated wines.

Eight aldehydes and two ketones were detected in all wines, but most of them were not significantly affected by the treatments and the OAVs of these compounds were relatively low, except for 3-methylbutanal (OAV around three). Only hexanal and benzaldehyde showed significant changes as they increased in 24H_CM + SF wine, indicating skin fermentation enhanced the formation of these compounds. Hexanal can be produced from oxidation of 1-hexanol as discussed by Campo *et al.* [34] or by enzymatic oxidation of C18 unsaturated fatty acids [29]. Benzaldehyde, which has a bitter almond character, is considered a sensory important benzene compound in wine [29]. In this study it was, however, found in a concentration much lower than the threshold (OAV < 0.01). According to Paloma *et al.* [5], shorter maceration had no significant effect on benzaldehyde in “Albillo” wine which is in agreement with our present study in 6H_CM wine. However, Vazquez *et al.* [3] observed that maceration and enzyme treatment largely increased benzaldehyde. The higher level of benzaldehyde in the 6H_CM + SF and 24H_CM + SF wines probably related to the levels of benzyl alcohol in the wines (Table 4). However, it can also originate from oxidation reactions by yeast on amino acids (phenylalanine), glycoside precursors, phenol compounds of the grape, or from some secondary compounds like phenyl acetic acid and p-hydroxybenzoic acid [35,36].

Terpenes are varietal compounds derived from the grapes where they often are found to be glycosidically-bound. They are usually associated with floral notes in wine aroma [29]. In this study, eight terpenes, including terpene alcohols and some of their oxides, were identified in the wines. Among them, linalool was dominating with the highest concentration and OAV in all wine samples. WC and DP wines had relatively low amount of linalool and hotrienol and their concentrations were below their thresholds. However, applied maceration caused an increase of these terpenes and this effect was more pronounced in skin fermented wines (6H_CM + SF and 24H_CM + SF). A good example would be the level of linalool which was significantly increased by SF treatment and reached the highest odor activity levels in the 24H_CM + SF wine. Moreover, (*E*)-β-ocimene and α-terpineol were significantly increased by SF but they had concentrations far below their thresholds. The presence of terpenes in wine is typically due to direct extraction of these compounds and the breakdown of glycoconjugates in the skin and in the solid parts of berry cells during the process of CM [2,9,18,22,36]. In addition, some of these predominant monoterpenes, such as linalool, hotrienol, and α-terpineol can also be formed from geraniol during vinification [37].

With regard to other compounds, two sulfur compounds (S-methyl thioacetate and 2-methyldihydro-3(2*H*)-thiophenone) and one C_13_-norisoprenoid (β-damascenone) were detected in all samples. Like some of the terpenes, the amount of β-damascenone was significantly increased by CM and SF treatments. This effect was in agreement with other studies [5,20]. The concentration of this compound exceeded its odor threshold in all samples. β-Damascenone can be formed by oxidative degradation of carotenoids during grape crushing, while a more probable source for damascenone in wine is acid-catalyzed rearrangement of C_13_ intermediates, with oxidation occurring in the grape during ripening [38].These precursors are, presumably, extracted to a greater extent during winemaking. β-Damascenone is an important trace compound with very low perception threshold (0.05 µg/L) in wine and gives wines fruity and honey odor [29].

**Table 4:** Volatile compounds quantified in the wines under different pre-treatments. Values are presented as averaged concentrations over two replicates (μg/L). For compounds exhibiting significant differences between treatments, values labelled with different letters.

Code	Compounds	Cal. LRI ^1^	Std. LRI ^2^	Odour Description ^3^	Concentration (μg/L)						Sig.	Odor	OAV ^5^
					DP	WC	6H_CM	24H_CM	6H_CM + SF	24H_CM + SF		Threshold (μg/L) ^4^	
*Esters*													
	*Ethyl esters*												
**e1**	**Ethyl propanoate**	**971**	**962**	**Fruit**	**202**	**221**	**199**	**183**	**213**	**209**	**ns**	**1800** **(1)**	**0.11–0.12**
**e2**	**Ethyl 2-methylpropanoate**	**969**	**969**	**Sweet, rubber**	**28.6 ^a,^^b^**	**36.9 ^a^**	**22.9 ^b,c^**	**24.3 ^b,c^**	**14.9 ^c^**	**16.7 ^c^**	**	**15** **(2)**	**0.99–2.5**
**e3**	**Ethyl butanoate**	**1038**	**1040**	**Apple**	**346**	**327**	**351**	**414**	**391**	**364**	**ns**	**20** **(2)**	**17–1**
**e4**	**Ethyl 2-methylbutanoate**	**1053**	**1058**	**Apple**	**1.79 ^a,b^**	**2.28 ^a^**	**1.34 ^b^**	**1.32 ^b^**	**0.97 ^b^**	**1.20 ^b^**	**	**1** **(2)**	**0.97–2.28**
**e5**	**Ethyl 3-methylbutanoate**	**1072**	**1079**	**Fruit**	**6.17 ^a,b^**	**7.90 ^a^**	**4.43 ^b,c^**	**4.15 ^c^**	**3.69 ^c^**	**4.51 ^b,c^**	*	**3** **(2)**	**1.2–2.6**
e6	Ethyl pentanoate	1153	1150	Yeast, fruit	0.420	0.342	0.437	0.501	0.387	0.455	ns	94 (3)	<0.001
e7	Ethyl (*E*)-butenoate	1178	1174	-	4.910	4.340	5.280	5.940	6.580	5.680	ns	-	-
**e8**	**Ethyl hexanoate**	**1263**	**1255**	**Apple peel, fruit**	**994**	**946**	**970**	**1070**	**950**	**870**	**ns**	**5** **(2)**	**170–210**
e9	Ethyl (*E*)-3-hexenoate	1327	1327	Pineapple, fruity	0.189 ^a,b^	0.161 ^a,^^b^	0.236 ^a^	0.175 ^a,b^	0.116 ^b^	0.980 ^b^	*	-	-
e10	Ethyl heptanoate	1354	1351	Fruit	0.100 ^c^	0.100 ^c^	0.142 ^b,^^c^	0.166 ^b^	0.179 ^b^	0.233 ^a^	***	220 (1)	<0.001
e11	Ethyl lactate	1353	1353	Fruit	59.0	55.1	51.3	35.4	54.7	62.8	ns	157,360 (1)	<0.001
**e12**	**Ethyl octanoate**	**1447**	**1450**	**Fruit, fat**	**617**	**544**	**648**	**743**	**661**	**560**	**ns**	**14** **(4)**	**39–53**
e13	Diethyl succinate	1691	1689	Wine, fruit	1600	1280	1390	850	1300	1820	ns	200,000 (1)	0.0043–0.010
e14	Ethyl 2-furoate	1641	-	-	7.25	7.50	6.22	6.43	8.09	8.85	ns	16,000 (5)	<0.001
**e15**	**Ethyl decanoate**	**1649**	**1651**	**Grape**	**180 ^a,b^**	**120 ^b^**	**174^ab^**	**231 ^a^**	**160 ^a,b^**	**110 ^b^**	**	**200** **(5)**	**0.55–1.2**
e16	Ethyl benzoate	1686	1690	Heavy, floral, fruity	7.23	9.58	16.0	8.76	8.12	10.7	ns	575 (5)	0.020–0.030
e17	Ethyl 9-decenoate	1703	1705	Fruit	-	-	0.0680	-	-	-	***	100 (6)	<0.001
e18	Ethyl dodecanoate	1854	1861	Leaf	3.50 ^a^	1.21 ^c^	3.50^a^	3.83 ^a^	2.95 ^b^	1.37 ^c^	*	500 (7)	<0.01
e19	Ethyl myristate	2071	2064	Floral, honey	0.202	0.188	0.200	0.128	0.0521	0.0700	ns	2000 (6)	<0.001
	*Total Ethyl esters*				4050	3560	3850	3590	3780	4050	ns		
	*Acetate esters*												
ac1	Propyl acetate	981	978	Sweet, fruity,	144	113	178	212	185	146	ns	4700 (1)	0.024–0.045
ac2	2-Methylpropyl acetate	1017	1018	Fruit, apple, banana	87.6	70.6	103	129	93.9	81.1	ns	1600 (8)	0.041–0.081
ac3	Butyl acetate	1078	1082	Pear	5.25	3.39	5.47	9.04	6.19	3.77	ns	1880 (1)	<0.001
**ac4**	**3-Methylbutyl acetate**	**1140**	**1142**	**Banana**	**10,300**	**8640**	**10,900**	**11,900**	**10,200**	**9110**	**ns**	**30** **(2)**	**290–360**
**ac5**	**Hexyl acetate**	**1299**	**1293**	**Fruit, herb**	**198**	**139**	**199**	**233**	**149**	**112**	**ns**	**1500** **(1)**	**0.075–0.15**
ac6	(*Z*)-3-Hexenyl acetate	1327	1328	Green, banana	0.0160	-	-	-	-	-	***	-	-
ac7	(*E*)-3-Hexenyl acetate	1333	1337	Sweet, Green, Sharp-fruity	4.97 ^a,^^b^	2.34 ^b^	4.45 ^a,^^b^	9.84 ^a^	5.41 ^a,^^b^	3.50 ^a,^^b^	*	-	-
ac8	Benzyl acetate	1749	1738	Fresh, boiled vegetable	-	-	0.214 ^b^	0.494 ^a^	0.430 ^a,b^	0.457 ^a^	**	2 (9)	<0.001
**ac9**	**Phenethyl acetate**	**1837**	**1835**	**Rose, honey, tobacco**	**361**	**255**	**332**	**358**	**351**	**334**	**ns**	**250** **(2)**	**1.0–1.4**
	*Total acetate esters*				11,100	9220	11,800	12,800	11,000	9790	ns		
*Other esters*													
oe1	Methyl hexanoate	1198	1196	Fruit, fresh, sweet	0.648 ^b^	0.648 ^b^	1.03 ^b^	1.73 ^a^	1.97 ^a^	2.04 ^a^	***	84 (10)	0.0077–0.024
oe2	Isopentyl butanoate	1289	1289	Sweet, apricot, banana	0.278	0.375	0.318	0.274	0.304	0.353	ns	-	-
oe3	Methyl octanoate	1400	1401	Orange	0.263 ^b^	0.224 ^b^	0.484 ^a,b^	0.841 ^a,b^	0.984 ^a^	0.872 ^a,b^	*	-	-
oe4	Methyl decanoate	1606	1608	Wine	0.0569 ^a,b^	0.0447 ^b^	0.0910 ^a,b^	0.171 ^a^	0.167 ^a^	0.136 ^a,b^	*	1200 (4)	<0.001
oe5	3-Methylbutyl octanoate	1668	1672	-	2.78	1.85	2.07	2.51	2.60	1.99	ns	125 (5)	0.015–0.022
oe6	Methyl salicylate	1800	1797	Pepper, mint	0.305 ^a,b^	0.130 ^b^	0.516 ^a,b^	0.641 ^a^	0.550 ^a^	0.500 ^a,b^	*	-	-
oe7	Ethyl phenylacetate	1806	-	Fruit, sweet	1.90	1.88	1.29	1.44	1.58	2.06	ns	-	-
	*Total other esters*				6.23 ^b,c^	5.15 ^c^	5.79 ^b,c^	7.61 ^a,b^	8.16 ^a^	7.95 ^a,b^	*		
*Alcohols*													
*Higher alcohols*													
alc1	1-Propanol	1041	1041	Alcohol, pungent	447 ^a,b^	357 ^a,b^	502 ^a^	296 ^a,b^	188 ^b^	217 ^b^	*	9000 (11)	0.024–0.056
alc2	2-Methyl-1-propanol	1104	1100	Wine, solvent, bitter	1020	1060	1210	1040	906	970	ns	40,000 (2)	0.023–0.030
alc3	1-Butanol	1164	1165	Medicine, fruit	251	216	267	348	227	196	ns	150,000 (4)	<0.001
**alc4**	**3-Methyl-1-butanol**	**1237**	**1238**	**Whiskey, malt, burnt**	**>10,000**	**>10,000**	**>10,000**	**>10,000**	**>10,000**	**>10,000**	**ns**	**30,000** **(2)**	**>0.33**
alc5	1-Pentanol	1279	1274	Balsamic	59.4 ^b^	58.8 ^b^	61.6 ^a,^^b^	64.8 ^a,^^b^	71.0 ^a,^^b^	98.5 ^a^	*	64,000 (1)	<0.001
alc6	1-Heptanol	1468	1471	Chemical, green	10.2	10.0	8.61	9.36	14.5	17.7	ns	-	-
alc7	2-Ethyl-hexanol	1502	1499	Rose, green	0.981	0.991	1.25	1.36	1.69	1.42	ns	8000 (6)	<0.001
alc8	1-Octanol	1570	1573	Chemical, metal, burnt	7.38	6.89	9.04	6.35	11.2	14.1	ns	900 (6)	0.01–0.016
alc9	Decanol	1774	1778	Fat	0.490	0.450	0.619	0.616	0.699	0.862	ns	400 (8)	<0.001
**alc10**	**2-Phenylethanol**	**1936**	**1935**	**Honey, spice, rose, lilac**	**18,500 ^a,b^**	**11,400 ^b,c^**	**17,200 ^b,c^**	**9590 ^c^**	**20,100 ^a,b^**	**24,400 ^a^**	*	**10,000** **(2)**	**1.1–2.4**
alc11	Benzyl alcohol	1896	1897	Sweet, flower	36.6 ^b^	37.2 ^b^	103 ^a^	105 ^a^	154 ^a^	151 ^a^	***	200,000 (4)	<0.001
*C6 alcohols*													
**alc12**	**1-Hexanol**	**1373**	**1372**	**Resin, flower, green**	**3250 ^a,b^**	**1730 ^b^**	**2900 ^a,b^**	**2340 ^a,b^**	**3520 ^a,b^**	**4280 ^a^**	*	**8000** **(2)**	**0.22–0.54**
alc13	(*E*)-3-Hexenol	1382	1386	Grass	16.6 ^a,b,c^	10.0 ^c^	12.3 ^b,c^	17.7 ^a,b^	20.2 ^a^	23.1 ^a^	**	150,000 (1)	<0.001
alc14	(*Z*)-3-Hexenol	1398	1390	Grass	1.86 ^c^	1.06 ^d^	2.44 ^c^	4.65 ^a^	3.15 ^b^	3.29 ^b^	***	400 (2)	<0.001
alc15	(*E*)-2-Hexenol	1421	1420	Green, leaf, walnut	0.502 ^c^	0.377 ^c^	1.12 ^b,c^	1.79 ^a,b^	1.92 ^a,b^	2.53 ^a^	**	15,000 (12)	<0.001
alc16	(*Z*)-2-Hexenol	1430	1430	Leaf, green, wine, fruit	0.321 ^c^	0.218 ^c^	1.10 ^b^	1.98 ^a^	1.85 ^a^	2.06 ^a^	***	-	-
*Aldehydes*													
**ald1**	**3-Methylbutanal**	**921**	**917**	**Malt**	**13.6**	**14.6**	**13.7**	**13.9**	**13.9**	**16.7**	**ns**	**4.6** **(1)**	**3.0–3.6**
**ald2**	**Hexanal**	**1087**	**1087**	**Grass, tallow, fat**	**1.40 ^b^**	**1.18 ^b^**	**1.25 ^b^**	**1.31 ^b^**	**1.76 ^a,b^**	**2.08 ^a^**	*	**9.1** **(13)**	**0.13–0.24**
ald3	Heptanal	1194	1192	Fat, citrus, rancid	0.472	0.439	0.406	0.543	0.477	0.554	ns	-	-
ald4	Octanal	1313	1311	Fat, soap, lemon, green	0.887	0.461	0.727	0.897	0.824	0.566	ns	-	-
**ald5**	**Nonanal**	**1405**	**1402**	**Fat, citrus, green**	**1.13 ^a,b^**	**0.789 ^b^**	**0.982 ^b^**	**1.45 ^a,b^**	**1.79 ^a^**	**1.31 ^a,b^**	*	**15** **(14)**	**0.053–0.12**
ald6	Decanal	1510	1511	Soap, orange peel, tallow	0.287	0.212	0.326	0.380	0.400	0.410	ns	10 (4)	0.021–0.041
ald7	Benzaldehyde	1541	1537	Almond, burnt sugar	3.84 ^b^	3.03 ^b^	4.82 ^b^	7.52 ^b^	12.5 ^b^	15.7 ^a^	**	2000 (11)	<0.01
	*Total aldehydes*				21.3 ^a,b^	20.8 ^b^	22.2 ^a,b^	26.0 ^a,b^	31.7 ^a,b^	37.4 ^a^	**		
*Ketones*													
k1	2-Heptanone	1192	1190	Soap	0.643	0.567	0.726	0.718	0.977	0.853	ns	-	-
k2	Acetoin	1310	1307	Butter, cream	2.98 ^b^	2.27 ^b^	3.23 ^b^	5.66 ^a^	1.89 ^b^	1.96 ^b^	***	150,000 (1)	<0.001
	*Total ketones*				3.63	2.84	3.96	6.38	2.87	2.81	ns		
*Terpenes*													
t1	Myrcene	1171	1170	Balsamic, must, spice	0.0667 ^a,^^b^	-	0.0764 ^a,^^b^	0.0904 ^a,^^b^	0.139 ^a^	0.174 ^a^	*	36 (13)	0.0040–0.012
t2	Limonene	1200	1200	Lemon, orange	0.256	0.280	0.278	0.354	0.634	0.596	ns	15 (4)	0.017–0.042
t3	(*E*)-β-Ocimene	1277	1277	Herbaceous, mild, citrus, sweet, orange	0.205 ^b^	0.212 ^b^	0.259 ^b^	0.333 ^a,^^b^	0.507 ^a^	0.567 ^a^	**	-	-
t4	*p*-Cymene	1291	1283	Lemon, fruity	0.0845	0.104	0.0427	0.0436	0.0706	0.0841	ns	11.4 (13)	<0.001
t5	Neroloxide	1482	1485	Oil, flower	5.69	5.89	6.80	6.83	6.75	8.93	ns	-	-
**t6**	**Linalool**	**1559**	**1560**	**Flower, lavender**	**7.85 ^b,c^**	**4.61 ^c^**	**11.8 ^b^**	**12.2 ^b^**	**12.40 ^b^**	**21.7 ^a^**	*	**15** **(8)**	**0.31–1.4**
**t7**	**Hotrienol**	**1623**	**1621**	**Hyacinth**	**2.52 ^c^**	**2.06 ^c^**	**7.37 ^b^**	**6.57 ^b^**	**7.55 ^b^**	**14.6 ^a^**	*	**100** **(13)**	**0.021–0.15**
t8	α-Terpineol	1712	1716	Oil, anise, mint	2.26 ^b,c^	1.30 ^c^	2.48 ^b,c^	3.40 ^b^	3.49 ^b^	5.76 ^a^	***	250 (8)	<0.001
	*Total terpenes*				18.9 ^c^	14.5 ^c^	29.1 ^b,c^	29.9 ^b,c^	31.5 ^b^	52.4 ^a^	***		
*Other compounds*													
ot1	*S*-Methyl thioacetate	1050	1050	Rotten, cooked vegetables	1.04 ^b^	0.980 ^b^	0.954 ^b^	0.443 ^c^	0.973 ^b^	1.41 ^a^	*	4500 (15)	<0.001
ot2	Dihydro-2-methyl-3(2*H*)-thiophenone	1546	1544	Cabbage, onion, must	3.33	3.08	1.98	1.38	5.48	5.00	ns	-	-
**ot3**	**β-Damascenone**	**1841**	**1844**	**Apple, rose, honey**	**0.310 ^c^**	**0.217 ^c^**	**0.583 ^b,c^**	**1.07 ^a,b^**	**1.56 ^a^**	**1.61 ^a^**	***	**0.05** **(2)**	**4.3–32**
	*Total other compounds*				4.68 ^b^	4.28 ^b^	3.52 ^b^	2.90 ^c^	8.02 ^a^	8.02 ^a^	**		

Significance levels: ns: not significant, *: *p* ≤ 0.05, **: *p* ≤ 0.01, ***: *p* < 0.001. Different letters in the same rows indicates significant differences according to Tukey’s HSD test; ^1^ The retention indices (RIs) of volatiles were calculated as the retention time of the volatiles normalized to the retention times of adjacently eluting *n*-alkanes (C6–C22) [39]; ^2^ Linear retention indices (LRI) calculated from authentic standard compounds analyzed on the same system; ^3^ Odour descriptions based on flavournet and pherobase online databases; ^4^ Odour threshold. (1) Etiévant, 1991 [40]; (2) Guth, 1997 [41]: (3) Takeoka, *et al.* 1995 [42]; (4) Gómez-Míguez, *et al.* 2007 [43]; (5) Ferreira, *et al.* 2000 [7]; (6) Tao, *et al.* 2010 [44]; (7) Moyano, *et al.* 2002 [45]; (8) Ferreira, *et al.* 2002 [46]; (9), Buttery, *et al.* 1982 [47]; (10) Takeoka, *et al.* 1991 [48]; (11) Peinado, *et al.* 2004 [13]; (12) Franco, *et al.* 2004 [49]; (13) Ahmed, *et al.* 1978 [50]; (14) Cullere, *et al.* 2004 [51]; (15) Moreira, *et al.* 2010 [52]. Odor threshold values were reported in wine-like matrices, except for (3), (9), (10), and (13) which were measured in water; ^5^ OAV: Odor activity value was calculated by dividing the concentration by the odor threshold value of the compound. Compounds with OAV > 0.1 are in bold. Only the loweset and highest values were showed in the table.

### 2.3. Sensory Evaluations

**Figure 1:**
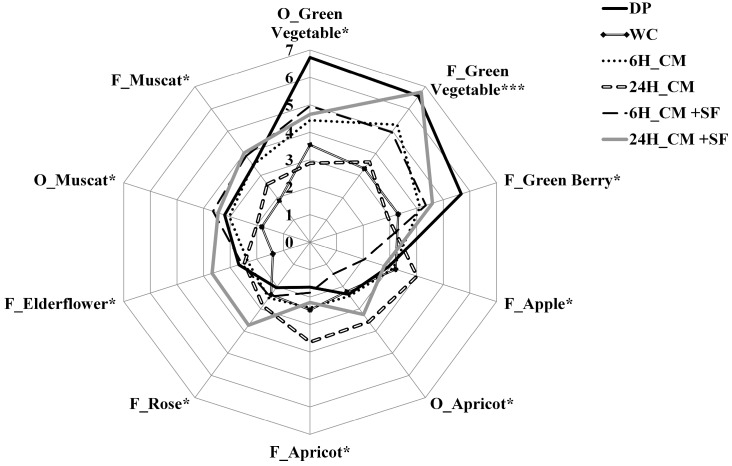
Sensory profile of six wines produced from different pre-fermentation procedures; Significant differences are shown: *: *p* ≤ 0.05, ***: *p* < 0.001; Suffix to the sensory descriptors indicates the type of assessment by panellists: O_: odor, F_: flavor.

According to the ANOVA, significant differences among samples were observed for the following 10 descriptors: O_Green Vegetable, O_Apricot and O_Muscat, F_Rose, F_Elderflower, F_Green Berry, F_Apple, F_Apricot F_Muscat, and F_ Green Vegetable. A spider plot was conducted using only the significant descriptors (Figure 1). In general, the wine vinified from WC showed the lowest scores of most attributes, indicating a weak aroma compared to the other wines. Two CM wines (6H_CM and 24H_CM) had higher values of descriptors but in different direction: 6H_CM wine was more linked with “F_Green Vegetable” while 24H_CM had a higher score of “F_Aprcot”. The DP wine was highly characterized by “F_Green Vegetable”, “O_Green Vegetable”, and “F_Green Berry”, whereas the most intense skin contact 24H_CM + SF wine had the highest values in the floral descriptors “F_Rose” and “F_Elderflower”. However, it should be noted that the 24H_CM + SF wine also had the highest value of “F_Green Vegetable”, indicating that combining maceration and skin fermentation increased floral notes but meanwhile exacted more green flavor from the grape skin. Furthermore, the effect of SF on perception of muscat characteristic was more pronounced since two samples with SF treatments (6H_CM + SF and 24H_CM + SF) were highly correlated with “F_Muscat” descriptor.

The influence of pre-treatment techniques on the sensory properties of wines has been previously reported. Palomo *et al.* [5] stated that “Albillo” wines produced with skin contact were higher in green apple, apricot, and peach notes than those of un-macerated wines. Similarly, Losada *et al.* [9] observed that white “Godello” wines made from must with 36 h macerated wines had the highest score for general quality. Longer maceration could have negative effect on wine quality, such as the increased extraction of phenolic compounds which result in more bitter taste [16]. In this work, samples were not significantly different in terms of bitter taste. This indicates that the relative low maceration temperature and short extraction time applied may only extract flavonoids in a lesser extent, herein limiting the bitterness in wine.

### 2.4. Relationship between Instrumental Analysis and Sensory Properties of Wines

**Figure 2:**
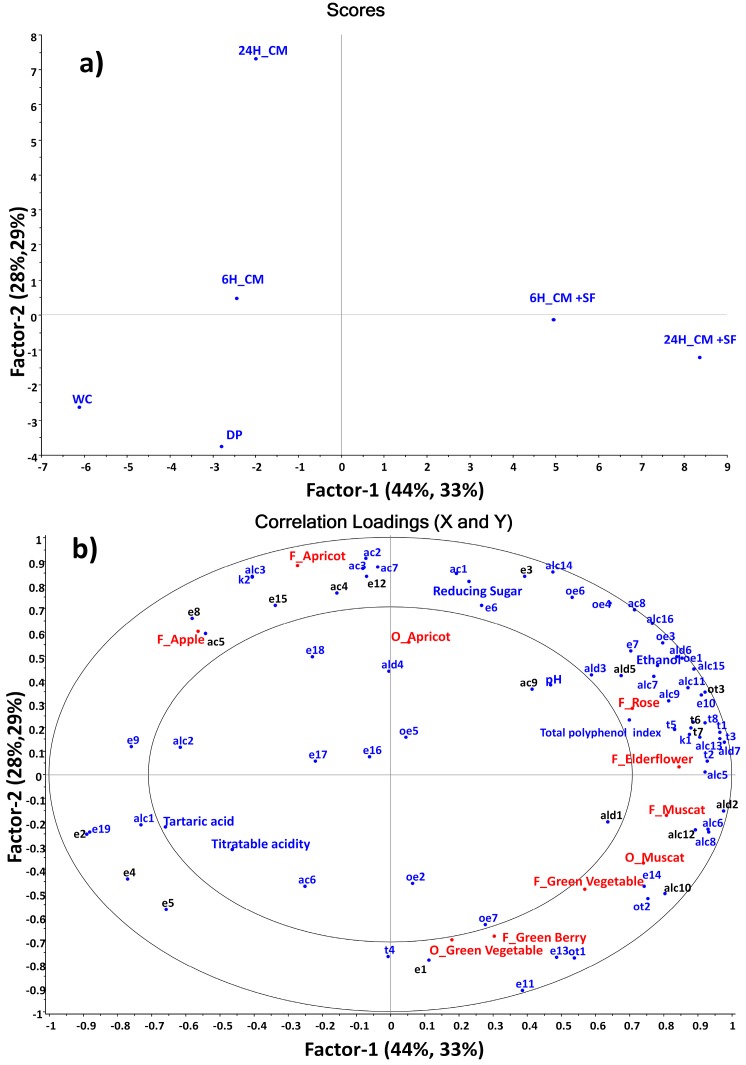
Partial Least Squares Regression (PLS) of wines correlation score (**a**) and loading plots (**b**) of the first two PCs. X-matrix: volatile compounds (**blue**, compounds with OAV > 0.1 are in **black** bold), chemical parameters; Y-matrix: sensory descriptors (**red**). The inner and outer ellipses represent R^2^ = 50% and 100%, respectively.

A Partial Least Squares (PLS) regression model was established to investigate the correlation between volatile compounds and chemical parameters (X-matrix) and significant sensory attributes (Y-matrix). The first two PLS components explained 72% of the total variance in the X-matrix and 62% for the Y-matrix. The score plot (Figure 2a) visualizes the differences and similarities of wines produced from various treatments. CM + SF wines (in the right part of the plot) were distinguished from the others in the left part of the plot mainly by PC1. 24H_CM wine was distinguished by PC2 while small variation between WC and DP wines was observed. In the loading plot shown in Figure 2b, most volatile compounds were located in the right side, positively linked with PC1, indicating that CM combined SF treatments resulted in high amount of most volatile compounds. Additionally, the CM + SF wines had higher scores in most sensory attributes studied. On the contrary, DP and WC wines were characterized by low levels of most aroma compounds while higher in “O_Green Vegetable” odor.

It is noticeable that the flavor attribute “F_Apricot” (mainly high in 24H_CM wine), was closely correlated with several fermentation-derived acetates, such as 3-methylbutyl acetate (OAV: 360, *r* = 0.82, *p* < 0.05), 2-methylpropyl acetate (*r* = 0.89, *p* < 0.05) and butyl acetate (*r* = 0.82, *p* < 0.05). Additionally, some ethyl esters of straight-chain fatty acids such as ethyl decanoate (OAV: 1.2) and ethyl octanoate (OAV: 53), normally described as having grape and fruity characteristics, were positively correlated to the “F_Apricot” (*r* = 0.75, *p* < 0.1, and *r* = 0.72, *p* < 0.1, respectively). Moreover, the “F_Apple” attribute was associated to ethyl hexanoate (OAV: 210, *r* = 0.85, *p* < 0.05) and hexyl acetate (OAV: 0.15, *r* = 0.81, *p* < 0.05) with fruity and apple peel characteristics. Among these esters that showed high correlation to fruity flavor, only ethyl decanoate was significantly different between samples. Some other acetates with low OAVs were also grouped close to the fruity flavor in 24H_CM wine (Figure 2b). This indicated that the perceived high fruity flavor in 24H_CM wine might be due to a combination of many esters. The contribution of esters to the fruity note of wine aroma was previously demonstrated by many studies [53,54,55,56]. Among their observations, Vilanova *et al.* [53] stated that the fruity flavor of Spanish “Albariño” wine was mainly related to esters, for instance, ethyl octanoate and ethyl hexanoate.

The “F_Rose” descriptor which characterized wines with SF treatment was correlated to volatile compounds typically derived from grapes, *i.e.*, nerol oxide (*r* = 0.96, *p* < 0.01), linalool (*r* = 0.92, *p* < 0.01) with OAV 1.4 and β-damascenone (*r* = 0.84, *p* < 0.05) with OAV 32 in 24H_CM + SF sample. These compounds were highly related to “F_Elderflower” as well. Both linalool and β-damascenone had high OAVs and showed large difference between samples, which thus could explain the high scores of “F_Rose” descriptor in the wines with SF treatment. Correlation between terpenes and floral descriptors has been reported in the literature [53] where the high amounts of monoterpenes mostly contributed to the floral characteristics of “Albariño” wine. Furthermore, Campo *et al.* [54] confirmed that linalool was an important contributor to the floral, sweet, and muscat descriptors in Spanish white wines. In one more recent study, Vilanova *et al.* [56] reported that the highest score of floral aroma perceived in “Blanco lexítimo” wine was attributed to the presence of linalool in this type of wine.

Few compounds were correlated with green vegetable (odor and flavor) in this study as most compounds had lower level in DP and WC wines, only (*Z*)-3-hexenyl acetate and 1-hexanol (*r* = 0.81, *p* < 0.05 and *r* = 0.87, *p* < 0.05, respectively) showed positive correlations. However, these two compounds did not present high odor activity in DP and WC wines, and the variation of 1-hexanol among different treatments was relative small (OAV between 0.22 and 0.54). Several studies illustrated that the green vegetable flavor was often related to 3-alkyl-2-methoxypyrazines, such as 3-isobutyl-2-methoxypyrazine (IBMP), 3-sec-butyl-2-methoxypyrazine (SBMP) and 3-isopropyl-2-methoxypyrazine (IPMP) even at very low concentrations (ng/L) [57,58,59]. In this study, compounds that were responsible for the green flavor might therefore not be detected. More sensitive techniques, like selected ion monitoring could be applied in future studies. Another potential explanation for more intense green flavor in the DP wine could be that lower concentrations of fruity compounds and, thus, less masking of green flavor, as described by Hein *et al.* [60].

## 3. Experimental Section

### 3.1. Chemical Standards

Chemical standards of volatile compounds were supplied by Sigma-Aldrich (St. Louis, MO, USA), Fluka (Madrid, Spain), Merck KGaA (Darmstadt, Germany). Ethanol (HPLC grade, 99.9%) and l(+)-tartaric acid (>99.5%) were from Sigma-Aldrich (Kiev region, Ukraine).

### 3.2. Grapes and Winemaking

Grapes of “Solaris” (Merzling × Gm 6493 (Zarya severa × Muscat Ottonel), cross made in 1975 Freiburg, Germany and varietal protection in 2001)) were cultivated in the field at the fruit genebank and research station “Pometet” (Copenhagen, Denmark) on 30th September in 2011 after 772 GDD (10 °C base T). The plants were trained in a VSP (vertical shoot position) system and the basic structure of the plant was a short stem with two short cordons (V shape) and pruned with a short two-budded stab and a long cane on each head. Planting distance is 1.5 m in the row and 3 m between rows.

Microvinification was performed in the cellar at “Pometet” in glass fermentation vessels of 5 L–10 L size. “Solaris” grapes were divided into six batches of 40 kg from which wines were made in duplicate. Different treatments were carried out for each batch as listed in Table 1. In the first batch, the grapes were pressed immediately after destemming and crushing, which was referred to DP wine. The second batch of grapes were pressed using the whole clusters without destemming (WC wine). The rest of the grapes were destemmed, crushed together and then divided into four portions and subjected either to cold maceration at 10 °C for different hours (6H_CM and 24H_CM wines), or to cold maceration combined with skin fermentation for 30 h in 10 L buckets covered with lid (6H_CM + SF and 24H_CM + SF wines). CO_2_-cover was used to protect the grape mash from oxidation during handling. Pressing of all treatments was done with a 20 L hydropress (Speidel, Ofterdingen, Germany). The cloudy juice of all samples was stored overnight in a cold room (3 °C) to allow settling and racked into new vessels to obtain clear juice for fermentation. The treatments with initiated SF continued fermentation directly after pressing. All batches were inoculated with the commercial yeast *Saccharomyces cerevisiae, bayanus* (Lalvin DV10TM from Lallemand) at 0.2 g/L, and moved to an 18 °C temperature-controlled room. All wines were fermented to dryness. After fermentation, wines were racked and 50 mg/L SO_2_ was added. Based on a preliminary bench test, wines revealed relatively high acidity. In order to minimize the dominance of sourness during sensory evaluation, between 0 and 2.5 g/L of acidity was removed using calcium carbonate (Table 2). The levels of deacidification applied were based on a pre-test in 100 mL scale on the newly fermented young wines. The pre-test included a sensory evaluation to determine the deacidification needed in each wine. Finally, the wines were moved to 3 °C for cold stabilization for four weeks after which the wines were racked again. Free SO_2_ was adjusted to 30–35 mg/L and the wines were bottled in 375 mL bottles and stored at 14 °C. The analyses were made after two months.

### 3.3. Chemical Parameters Analysis

°Brix, potassium, ammonia, α-amino nitrogen, ethanol, pH, volatile acidity, tartaric acid, malic acid, fructose, total phenolic index (Folin-Ciocalteu index), glycerol, and reducing sugar were determined using a WineScan instrument (WineScan™ FT120: Foss, Hillerød, Denmark) based on Fourier Transformed Infrared Spectrophotometry. The calibration of the instrument was validated by parallel measurements of chemical parameters of 75 Danish wines, of which 10 commercial Solaris wines. A good agreement was found with R^2^ ranged from 0.9 to 0.96 between traditional methods and WineScan method for the different parameters. Therefore it was decided to use WineScan to measure the chemical parameters of the experimental wines. Titratable acidity was determined by titrating with 0.33 M NaOH solution to a pH end-point of 8.2 and expressed in g/L of tartaric acid equivalents. All samples were analyzed in duplicate.

### 3.4. Volatile Compound Analysis

Dynamic headspace sampling (DHS) was applied to extract aroma compounds. Twenty mL of each wine was transferred to a 100 mL flask and 1 mL of 4-methyl-1-pentanol (50 mg/L, Aldrich, Steinheim, Germany) was added as an internal standard. The sample was equilibrated to 37 ± 1 °C and then purged with a nitrogen flow of 100 mL/min for 20 min with magnetic stirring at 200 rpm. The volatile compounds were trapped on a Tenax-TA tube (200 mg, mesh size 60/80, Buchem BV, Apeldoorn, The Netherlands). After purging, the tube was dried directly with nitrogen for 10 min to remove excess water. System control samples were run by using an empty flask at the same condition as the wine samples. All samples were analyzed in duplicate.

The collected volatile compounds were analyzed according to methodology developed in our lab [61] by a thermal desorption (ATD 30, PerkinElmer, Norwalk, CT, USA) gas chromatography mass spectrometry system (GC-MS, 7890A GC system interfaced with a 5975C VL MSD with Triple-Axis detector from Agilent Technologies, Palo Alto, CA, USA) equipped with a DB-Wax column (J & W Scientific 30 m × 0.25 mm × 0.25 mm). GC-MS data was analyzed using MSD Chemstation G1701EA (Version E.01.00.237, Agilent Technologies Inc., Palo Alto, CA, USA). The column pressure was held constant at 2.4 psi resulting in an initial flow rate of approximately 1.2 mL/min using hydrogen as carrier gas. The column temperature was kept at 30 °C for 10 min, increased at 8 °C/min to 240 °C, and finally kept isothermal for 5 min. The mass detector conditions were: electron-impact ionization (EI) at 70 eV, a scan range of 15–300. The MS transfer line was set to 225 °C. The identification of volatile compounds was achieved by matching their mass spectra with those of a standard library (Wiley275.l, HP product no.G1035A) and with those of pure authentic standards. To further verify the identification, linear retention indices (LRI) were calculated with a homologous series of alkanes (C5–C22) (Hewlett-Packard Co, Avondale, PA, USA) and compared to those of the authentic standards and/or literature values (Table 4).

Quantification of the volatile compounds was carried out from calibration curves obtained by each compound in synthetic wine. The synthetic wine was a 12% (*v*/*v*) ethanol solution in water containing 3.5 g/L of l-(+)-tartaric acid and the pH was adjusted to 3.4 with 5 M NaOH. Individual standard compounds were accurately weighed and dissolved in ethanol (99.9%) and then combined and diluted with synthetic wine to obtain a relevant range of concentrations. One mL of internal standard was added to the stock solution of each calibration level that was further extracted and analyzed under the same conditions as the wine sample. Standards were analyzed in triplicate.

### 3.5. Sensory Evaluation

A descriptive sensory analysis was conducted by a panel of eleven trained winemakers (four females and seven males, graduating from “Bayerischen Landesanstalt für Weinbau und Gartenbau” in Veitshöchheim, Germany). A set of sensory descriptors, including nine odors (O_Rose, O_Elderflower, O_Green Vegetable, O_Green Berry, O_Apple, O_Apricot, O_Pineable, O_Citrus, O_Muscat), eleven flavours (F_Rose, F_Elderflower, F_Green Vegetable, F_Green Berry, F_Apple, F_Apricot, F_Pineapple, F_Citrus, F_Muscat, F_Sulfurous, F_Body), three tastes (T_Bitter, T_Sourness, T_Sweetness) and astringency was generated by the panel. Panelists were then asked to score the wines on an unstructured, 15-cm linear scale using the attribute list. 30 mL of each wine was served in standard wine-tasting glasses coded with three digit numbers. The samples were presented in randomized order to minimize systematic carry-over effects. Cold water was used as palate cleansing. All the evaluations were done in ventilated tasting rooms at 20 °C in duplicate.

### 3.6. Statistical Analyses

One-way analysis of variance (ANOVA) was applied to concentrations of volatile compounds measured from fermentation duplicates by JMP 9.0.2 (SAS Institute, Cary, NC, USA) using sample as fixed effect and post-hoc by Tukey’s HSD test. Two-way ANOVA was performed on sensory data (products and assessors as the fixed factors) using PanelCheck V1.4.0 (Matforsk, Tromsø, Norway). Correlations between chemical composition (X-matrix) and sensory attributes (Y-matrix) were investigated by partial least squares regression (PLS) analysis using Unscrambler version 9.7 (CAMO ASA, Oslo, Norway).

## 4. Conclusions

The current study was the first to investigate the influence of different pre-fermentation treatments on the properties of “Solaris” wines. Cold maceration (CM) combined skin fermentation (SF) reduced acidity, leading to wines with slightly higher ethanol, glycerol, and total polyphenol index. SF combined with 24 h of CM treatment tended to make “F_Rose” and “F_Elderflower” notes more pronounced in the final wines. These treatments also enhanced formation/extraction of some volatile compounds such as C-6 alcohols, linalool and β-damascenone, while various effects were found on esters depending on the compounds. The pure CM treatment reduced the intensity of “F_Green Vegetable” and “F_Green Berry” sensory characteristics and enhanced the “F_Apricot” and “F_Apple” notes. Finally, whole cluster press (WC) and direct press (DP) generally resulted in wines with low content of aroma compounds and, thus, a weak flavor. The PLS regression revealed that fruity flavor was closely related to the combination of esters, while “F_Rose” and “F_Elderflower” were correlated to β-damascenone and linalool. In conclusion, when making “Solaris” wines, proper pre-treatments, such as cold maceration and short fermentation on skin are capable to modify the volatile profile of wines, which in turn can diversify the wine style and influence the quality.
